# Silent Entry, Rapid Demise: Fulminant Clostridium septicum Necrotizing Fasciitis in an Immunosuppressed Host

**DOI:** 10.7759/cureus.89889

**Published:** 2025-08-12

**Authors:** Hugo Inácio, Rita Jorge, Carla A Costa, Ângela Simas

**Affiliations:** 1 Intensive Care Unit, Unidade Local de Saúde Loures-Odivelas, Hospital Beatriz Ângelo, Lisboa, PRT

**Keywords:** brest cancer, clostridium septicum, gas embolism, immunocompromised patient, necrotizing fasciitis

## Abstract

*Clostridium septicum* is a rare but highly virulent anaerobic pathogen associated with spontaneous gas gangrene and necrotizing soft tissue infections, particularly in immunocompromised individuals and patients with underlying malignancies. Its rapid progression and subtle early symptoms make timely diagnosis and treatment extremely challenging.

We report the case of a 39-year-old woman with metastatic neuroendocrine breast carcinoma and diffuse bone marrow infiltration who developed fulminant necrotizing fasciitis due to *C. septicum*. Two weeks after initiating systemic therapy with a selective cyclin-dependent kinase (CDK) 4/6 inhibitor, she presented to the emergency department with rapidly progressing right thigh pain, hemodynamic instability, and laboratory evidence of severe pancytopenia and rhabdomyolysis. Computed tomography revealed extensive soft tissue emphysema extending into the retroperitoneum, along with intravascular gas in the iliac veins, inferior vena cava, and right ventricle. Despite prompt initiation of broad-spectrum antibiotics, vasopressor support, and preparation for emergency surgical debridement, the patient suffered a sudden cardiac arrest and died within hours of admission. Blood cultures later confirmed *C. septicum* infection.

This case underscores the aggressive course of *C. septicum* infections and their strong association with malignancy and immunosuppression. The combination of soft tissue emphysema and intravascular gas suggests gas embolism as a contributing factor to the patient’s abrupt clinical decline.

Clinicians must maintain a high index of suspicion for *C. septicum* infection in patients with localized pain and systemic deterioration, especially in immunocompromised hosts. Early recognition and multidisciplinary management are essential, although outcomes may remain unfavorable despite timely and aggressive intervention.

## Introduction

Necrotizing fasciitis (NF) is a rare and severe bacterial infection of the soft tissues, characterized by extensive tissue destruction. It primarily affects the deep fascia and subcutaneous tissues but may also extend to the skin and underlying muscles, particularly in advanced or untreated cases [1-7]. The clinical presentation is distinguished by rapid progression, intense pain, severe systemic manifestations, and high mortality, especially in the absence of early diagnosis and intervention [4]. Although often associated with penetrating trauma or surgical wounds, NF can occur in the absence of obvious lesions and is then classified as spontaneous or atraumatic [6,7].

Among the etiological agents, species of the genus *Clostridium* are particularly noteworthy, known for their high virulence and ability to cause fulminant infections, such as gas gangrene (or clostridial myonecrosis) [4].

*Clostridium perfringens* is frequently implicated in extensive traumatic infections, while *Clostridium septicum* can trigger the infectious process spontaneously or after minimal trauma [6,7]. A distinctive and clinically significant feature of *C. septicum* infections is their strong association with underlying pathology, particularly hematological and gastrointestinal tract malignancy, as well as with immunosuppressive states [1,3,5,8]. It is believed that disruption of the intestinal mucosal barrier or host immunosuppression can favor the translocation and hematogenous dissemination of this opportunistic microorganism [6,8].

Infection by *C. septicum* is characterized by the production of potent exotoxins, such as alpha toxin, which promote rapid tissue destruction and vascular occlusion. These toxins induce muscle necrosis and microvascular thrombosis, leading to extensive tissue damage and a high risk of septic shock [6,9]. In addition to direct injury to muscle and connective tissue cells, alpha toxin compromises microvascular perfusion by promoting platelet aggregation, leukocyte activation, and endothelial injury, further exacerbating tissue ischemia. The resulting hypoxic and acidic environment favors bacterial proliferation and sustained toxin production [6].

The typical clinical presentation includes disproportionate, intense pain, edema, erythema, formation of hemorrhagic bullae, subcutaneous crepitus, and rapid progression to shock and multiorgan failure. In cases of spontaneous gas gangrene by *C. septicum*, the infection can start insidiously but evolve quickly to a fulminant condition [1,7].

The mortality associated with *C. septicum* infection is particularly high, estimated between 67% and 100%, with many deaths occurring within the first 24 hours of symptom onset [7]. The overall incidence of *Clostridium* bacteremia in the general population is low (approximately 4.9 per 100,000 persons/year, according to population studies), with *C. septicum* representing a minority of these cases but with a strong association with neoplasms, as approximately 71% of cases of spontaneous gas gangrene by *C. septicum* occur in patients with an underlying malignant disease [6,10]. Early recognition, administration of broad-spectrum antibiotics with antitoxin activity, and urgent surgical debridement are the cornerstones of treatment, although they often prove insufficient due to the infection’s extremely rapid progression [1,6,8,11].

We present a case that underscores the diagnostic and therapeutic challenges associated with *C. septicum* infections. It highlights the need for a high index of suspicion in immunocompromised patients presenting with rapidly progressive soft tissue infections.

## Case presentation

A 39-year-old woman, previously healthy, was diagnosed in August 2024 with an invasive carcinoma of the right breast, exhibiting a poorly differentiated neuroendocrine phenotype. Immunohistochemistry revealed strong estrogen receptor positivity (ER 100%), negative progesterone receptors (PR), HER2 negativity, and a high proliferation index with Ki-67 at 90%. Staging workup confirmed cT3N3M1 disease, with extensive bone and bone marrow metastases on computed tomography (CT) and bone scintigraphy.

Since diagnosis, she had been experiencing neuropathic low back pain radiating to the lower limbs, accompanied by paresthesia, requiring opioids, non-steroidal anti-inflammatory drugs (NSAIDs), and duloxetine for symptom control.

On November 18th, 2024, she initiated systemic treatment with letrozole, ribociclib, and goserelin. At treatment onset, laboratory tests showed elevated CA 15.3 levels (270 U/mL), moderate anemia (hemoglobin 8.0 g/dL), thrombocytopenia (90 × 10⁹/L), normal leukocyte count, and preserved renal function (estimated glomerular filtration rate [eGFR] 81 mL/min/1.73 m²) (Table 1). Bone marrow aspiration was not performed at this stage.

Fifteen days later, on December 3rd, routine laboratory follow-up revealed newly developed pancytopenia: hemoglobin 5.7 g/dL, leukocyte count 2.47 × 10⁹/L (neutrophils 1.01 × 10⁹/L), and platelet count 88 × 10⁹/L (Table 1).

**Table 1:** Evolution of laboratory parameters with corresponding reference ranges

Parameter	Nov 18, 2024	Dec 3, 2024	Dec 9, 2024	Reference Range	Unit
Hemoglobin	8.0	5.7	5.4	12.0 – 15.0	g/dL
Leukocytes	8.08	2.47	0.34	4.5 – 11.0	×10⁹/L
└ Absolute Neutrophils	4.27	1.01	0.09	2.0 – 8.5	×10⁹/L
Platelets	90	88	8	150 – 450	×10⁹/L
Creatinine	0.89	1.03	1.37	0.51 – 0.95	mg/dL
└ eGFR (CKD-EPI)	81	68	48	>90	mL/min/1.73 m²
Potassium (K⁺)	3.0	3.5	6.2	3.5 – 5.1	mEq/L
Total Calcium	8.5	8.5	6.6	8.6 – 10.0	mg/dL
Phosphate	—	—	12.6	2.5 – 4.5	mg/dL
Creatine Kinase (CK)	—	—	21,774	<170	U/L
Myoglobin	—	—	>30,000	25 – 58	ng/mL
LDH	571	319	1,922	135 – 214	U/L
INR	—	—	2.10	0.80 – 1.20	—
APTT	—	—	46.9	25.1 – 36.5	seconds
Fibrinogen	—	—	1.4	2.0 – 4.0	g/L
CRP (C-Reactive Protein)	—	—	62.4	<5.0	mg/L
CA 15.3	270	190	—	<28.5	U/mL

Three weeks later, on December 9th, the patient presented to the emergency department with a two-day history of intense fatigue and severe pain in the right thigh. She denied fever. On initial assessment, the patient was in pain and afebrile, with a blood pressure of 171/73 mmHg, a heart rate of 114 bpm, and an oxygen saturation of 97% on room air. Physical examination revealed significant swelling of the right lower limb, predominantly the thigh, with severe tenderness on palpation and passive mobilization.

Laboratory investigations revealed severe pancytopenia: hemoglobin 5.4 g/dL, leukocytes 0.34 × 10⁹/L (neutrophils 0.09 × 10⁹/L), and platelets 8 × 10⁹/L. Other findings included acute kidney injury (creatinine 1.37 mg/dL; eGFR 48 mL/min/1.73 m²), hyperkalemia (6.2 mEq/L), hypocalcemia (6.6 mg/dL), hyperphosphatemia (12.6 mg/dL), and marked rhabdomyolysis (creatine kinase [CK] 21,774 U/L, myoglobin >30,000 ng/mL, lactate dehydrogenase [LDH] 1922 U/L). C-reactive protein (CRP) was markedly elevated at 62.4 mg/L (reference value <5.0 mg/L). Coagulation tests were abnormal: INR 2.10, activated partial thromboplastin time (aPTT) 46.9 seconds, and fibrinogen 1.4 g/L (Table 1). 

The patient received transfusions of three units of packed red blood cells, three units of fresh frozen plasma, and three platelet pools. Intravenous fluid resuscitation and electrolyte correction were promptly initiated. Broad-spectrum empiric antibiotic therapy was started immediately after blood cultures were drawn, consisting of piperacillin-tazobactam (4.5 g), vancomycin (2 g), and clindamycin (900 mg), in the context of a severe soft tissue infection in a profoundly immunocompromised patient.

She was evaluated by the general surgery team, who, given the extent of lower limb edema and the alarming laboratory findings, requested urgent CT imaging to investigate potential underlying causes and to assess the need for surgical intervention.

**Figure 1:**
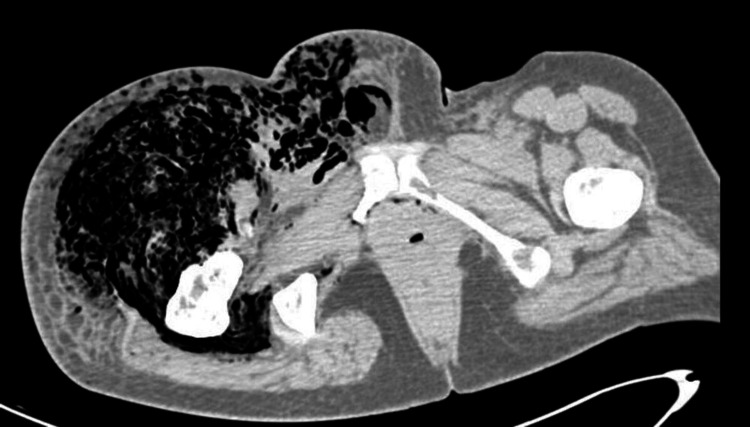
Axial CT scan of the pelvis showing extensive soft tissue edema and widespread gas collections dissecting through fascial and muscular planes in the right hemipelvis, indicative of tissue necrosis and gas-forming infection. The left hemipelvis shows preserved soft tissues.

**Figure 2:**
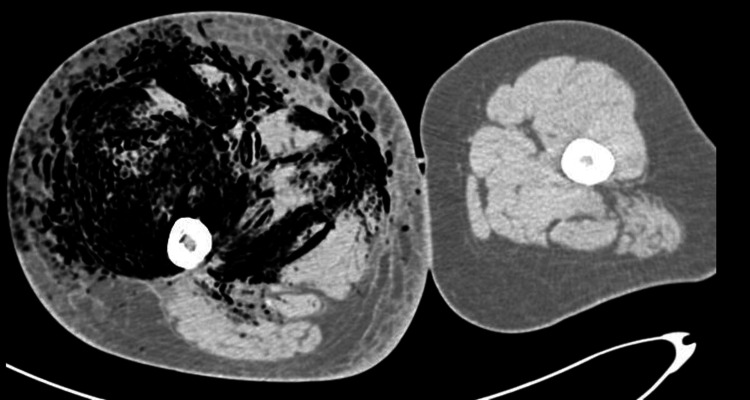
Axial CT scan of the right thigh showing severe soft tissue edema and extensive gas collections dissecting through subcutaneous fat and muscular compartments, consistent with necrotizing fasciitis. There is significant swelling and disruption of normal fascial planes.

**Figure 3:**
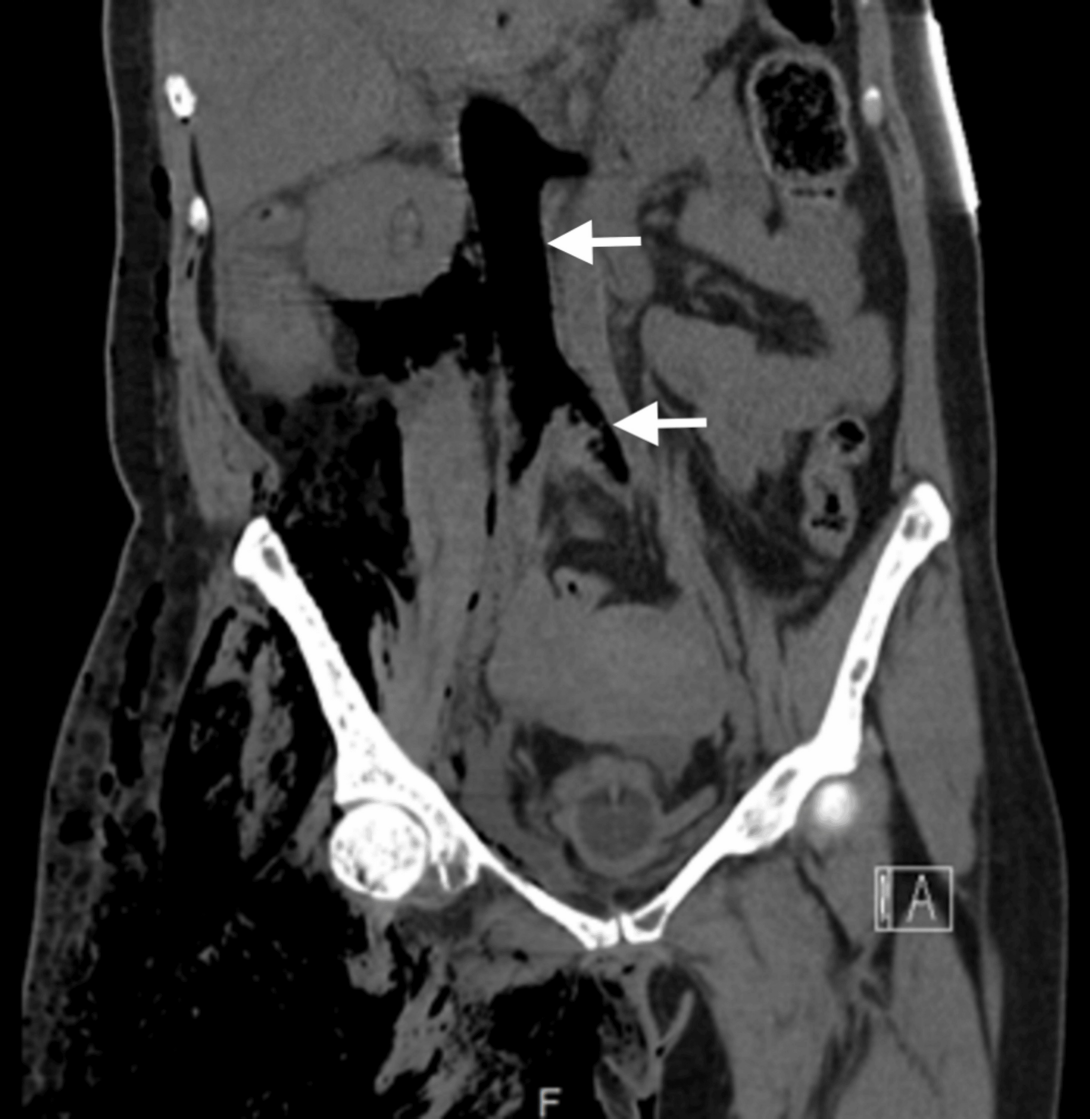
Coronal CT scan of the abdomen and pelvis showing extensive gas dissection from the right thigh into the retroperitoneum, with intravascular gas in the iliac venous system and inferior vena cava (white arrows).

**Figure 4:**
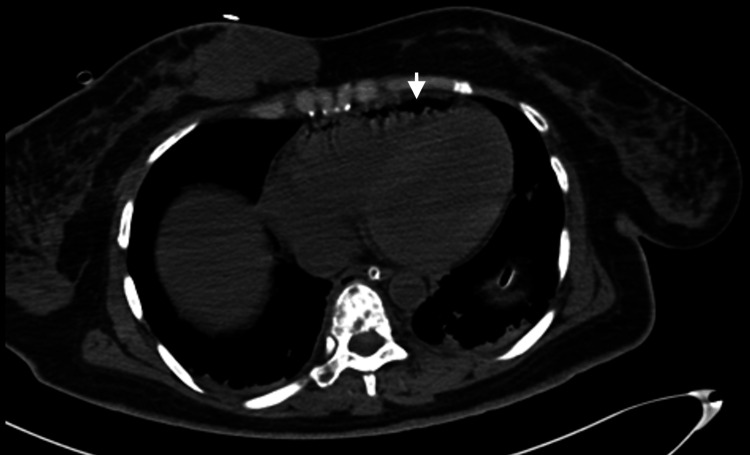
Axial CT scan of the chest with intracardiac gas (white arrow).

CT revealed extensive necrotizing fasciitis of the right thigh, with subcutaneous and muscular emphysema (Figures 1, 2) extending into the retroperitoneum (Figure 3). Intravascular air was identified in the external iliac vein, common iliac veins, inferior vena cava (Figure 3), and even the right ventricle (Figure 4). Additional findings included gas in peripheral branches of the portal venous system, renal perfusion abnormalities, thickening of the ascending colon, and bone lesions suggestive of metastasis.

Following the CT scan, and about six hours after hospital admission, the patient experienced abrupt clinical deterioration. On reassessment, she was hemodynamically unstable: blood pressure 84/52 mmHg, heart rate 147 bpm, afebrile (tympanic temperature 36.8°C), and tachypneic, with an oxygen saturation of 96% on room air. Her right thigh was markedly swollen with palpable crepitus extending from the thigh to the foot. Peripheral pulses were barely palpable. Arterial blood gas analysis demonstrated severe metabolic acidosis with hyperlactatemia and electrolyte abnormalities (pH 7.02, pCO₂ 33 mmHg, pO₂ 100 mmHg, HCO₃⁻ 8.5 mmol/L, lactate 15 mmol/L, sodium 133 mEq/L, potassium 5.3 mEq/L). Point-of-care cardiac ultrasound was limited by poor acoustic windows. Although suggestive of preserved biventricular function, no further structural or hemodynamic assessment could be reliably obtained.

Due to rapid clinical deterioration, the patient was intubated and mechanically ventilated, with adequate ventilatory adaptation under sedation and analgesia using propofol and fentanyl. Fluid resuscitation was intensified, with a total of 3.5 liters of crystalloids administered. Sodium bicarbonate 8.4% was administered for severe acidosis. Hyperglycemia (~300 mg/dL) was observed, without significant ketonemia (0.1 mmol/L). Norepinephrine was initiated and titrated up to 0.4 µg/kg/min to maintain a mean arterial pressure (MAP) >65 mmHg.

Despite aggressive resuscitation and full transfusional support, the patient progressed to refractory septic shock requiring escalating vasopressor support. Hydrocortisone and vasopressin were added when norepinephrine requirements exceeded 0.5 µg/kg/min.

She was transferred to the operating room for emergent surgical debridement. However, shortly after arrival, approximately eight hours after hospital admission, she suffered a cardiac arrest in asystole. Resuscitation efforts were unsuccessful, and the patient was pronounced dead.

Blood cultures later identified *C. septicum*. An autopsy was not performed at the request of the family due to religious reasons.

## Discussion

This report describes the rapidly fatal course of a 39-year-old woman with a poorly differentiated neuroendocrine-type breast carcinoma, strongly estrogen receptor-positive and HER2-negative, with diffuse bone and bone marrow metastases. Shortly after the initiation of treatment with ribociclib, a selective cyclin-dependent kinase (CDK) 4/6 inhibitor, the patient developed severe iatrogenic neutropenia, which likely facilitated the onset of an opportunistic infection caused by *C. septicum*, ultimately complicated by venous gas embolism.

At presentation, the patient exhibited diffuse bone metastases, anemia (Hb 8.0 g/dL), and moderate thrombocytopenia (90 × 10⁹/L). These findings, together with bone scintigraphy, suggested osteomedullary infiltration, although no myelogram was performed for histological confirmation. On November 18th, 2024, treatment was initiated with letrozole, ribociclib, and goserelin. Over the subsequent two weeks, the patient experienced progressive cytopenia deterioration, culminating in severe pancytopenia, a condition consistent with the combined effects of bone marrow infiltration and the hematologic toxicity of ribociclib, a drug commonly associated with neutropenia [12,13].

The precise frequency of diffuse bone marrow infiltration by poorly differentiated neuroendocrine breast tumors remains unknown due to the rarity of such cases. While most reports emphasize the presence of bone metastases, diffuse marrow infiltration can occur, particularly in advanced disease stages, resulting in cytopenias, most notably anemia and thrombocytopenia [14,15]. Therefore, in patients with neuroendocrine breast carcinoma who develop unexplained cytopenias, as illustrated in this case, bone marrow infiltration should be considered. Diagnostic confirmation through myelography and bone biopsy is recommended to guide appropriate management.

The hematological toxicity of ribociclib is well established, with neutropenia being the most common treatment-related adverse event. This effect typically manifests within the first few weeks of treatment, as observed in the present case [12,16]. Depending on the degree of hematological recovery and the recurrence of cytopenias, dose reduction or, in some cases, permanent discontinuation of the drug may be required. Importantly, the myelosuppressive effects of ribociclib are generally reversible with appropriate dose adjustments or treatment interruption [16]. Initiating ribociclib in patients with an already compromised hematopoietic reserve warrants careful consideration, as the resultant immunosuppression can increase susceptibility to severe infections with significant mortality risk.

In this case, the profound pancytopenia, resulting from both disease-related marrow infiltration and treatment-induced myelosuppression, created a severely immunocompromised state that significantly increased the patient's susceptibility to opportunistic infections, including rare and fulminant pathogens such as *C. septicum*.

Timely recognition of NF is essential due to its aggressive progression and high mortality. A high index of clinical suspicion is often the cornerstone of early diagnosis. The Infectious Diseases Society of America (IDSA) emphasizes that certain features, such as severe pain out of proportion to physical findings, rapid symptom progression, and early signs of systemic toxicity, are hallmark indicators of NF, even in the absence of fever or when cutaneous signs are subtle. The typical swift evolution toward hemodynamic instability also helps distinguish it from more indolent, non-necrotizing soft tissue infections [1,4,8].

In selected cases where emergent surgical indications are not immediately evident, prompt imaging after presentation may help identify deep tissue involvement and accelerate surgical decision-making. Early engagement of all relevant specialties, including surgery, infectious diseases, and critical care, can promote coordinated management and may contribute to improved patient outcomes.

In this case, the diagnosis of NF was not established in a timely manner. Although crepitus was not documented on the initial physical examination of the right thigh, it was likely already present. The lack of fever in a profoundly immunocompromised patient likely further obscured the clinical picture. At that point, signs suggestive of developing compartment syndrome were probably already present, which could have warranted urgent surgical intervention without the need for prior imaging. Still, given the fulminant progression of the disease, the outcome may have remained unchanged despite earlier recognition and intervention.

The isolation of *C. septicum* from blood cultures ultimately confirmed it as the causative agent of NF with vascular gas invasion. *C. septicum* is a gram-positive, anaerobic, spore-forming bacillus with a predilection for hypoxic or ischemic tissues. It can cause fulminant infections, often in the absence of overt cutaneous manifestations [2-6,11]. This organism is frequently linked to underlying malignancies, particularly colorectal cancer, and to states of profound immunosuppression, including neutropenia secondary to chemotherapy [4-9,11], as exemplified in the present case.

Optimal antimicrobial management of severe *C. septicum* infections typically includes a combination of penicillin and clindamycin. Nevertheless, IDSA guidelines recommend initiating broad-spectrum empirical therapy, such as vancomycin in combination with piperacillin-tazobactam, ampicillin-sulbactam, or a carbapenem, pending microbiological identification. Clindamycin is specifically emphasized for its ability to inhibit bacterial toxin synthesis and for its activity independent of the bacterial growth phase, a particularly important feature in clostridial infections where toxin-mediated damage is a major contributor to disease severity and mortality. Urgent surgical exploration and debridement remain cornerstone interventions and should be pursued without delay, in parallel with antimicrobial therapy [1,3,5,6,11].

The radiological finding of parietal thickening of the ascending colon, although nonspecific, raises the possibility of bacterial translocation and may have constituted the portal of entry for *C. septicum*. The presence of gas at the periphery of the portal system further supports an enteric origin. The possibility of colonic metastasis or even a subclinical primary intestinal focus cannot be excluded. Although rare, colonic metastases from breast carcinoma have been described in the literature, particularly in association with more aggressive histological subtypes such as invasive lobular carcinoma. To date, however, no cases with neuroendocrine differentiation have been reported in the cited literature [17]. Regardless of the exact etiology, in the setting of profound immunosuppression, a previously compromised colonic mucosa may become susceptible to translocation of highly invasive anaerobic microorganisms such as *C. septicum*, thereby facilitating the development of fulminant infections [6,8].

Vascular involvement was particularly prominent in this case. CT demonstrated the presence of gas within the iliac venous system, the inferior vena cava, and the right ventricle. These findings are indicative of venous gas embolism, a rare but recognized complication in the setting of *C. septicum* infections, attributed to the prolific gas production by this anaerobic bacterium [2]. Under conditions of low venous pressure, gas may enter the circulation, accumulate in the right heart chambers, and subsequently travel to the pulmonary artery, resulting in a massive gas embolism. This can precipitate sudden hemodynamic collapse by obstructing right ventricular outflow, ultimately leading to cardiorespiratory arrest [2]. While refractory septic shock is a common cause of mortality in such cases, the radiological evidence of vascular gas invasion provides a pathophysiologically plausible explanation for the abrupt clinical deterioration observed in this patient.

This case highlights the critical importance of a thorough hematological risk assessment prior to initiating potentially myelosuppressive therapies, particularly in patients with suspected active bone marrow infiltration. In such scenarios, confirmation of marrow involvement through myelogram and/or bone biopsy should precede therapeutic decisions, allowing for a careful risk-benefit evaluation.

Simultaneously, it underscores the need to maintain a high index of suspicion for *C. septicum* infections in oncology patients presenting with intense localized pain and signs of sepsis, particularly when deep tissue involvement and soft tissue emphysema are clinically suspected and subsequently confirmed by imaging studies.

## Conclusions

*C. septicum* is a rare but highly aggressive pathogen, frequently associated with underlying malignancies. This case illustrates the fulminant progression of *C. septicum* NF in a severely immunocompromised oncology patient, highlighting the importance of early recognition and prompt multidisciplinary intervention. The absence of trauma should not preclude the diagnosis in high-risk individuals, particularly those with pancytopenia or bone marrow involvement.

Despite early antimicrobial therapy and intensive supportive care, the prognosis remains poor, underscoring the need for careful risk-benefit assessment when initiating immunosuppressive treatment in patients with limited hematologic reserve. In this case, three critical factors converged: profound immunosuppression, a likely enteric portal of entry, and hematogenous gas dissemination. Together, they led to a fatal outcome and highlight an important opportunity for reflection on the clinical management of complex oncology patients at high risk for severe infections.
